# Non-Invasive Drug Delivery Systems

**DOI:** 10.3390/pharmaceutics13050611

**Published:** 2021-04-23

**Authors:** Driton Vllasaliu

**Affiliations:** School of Cancer & Pharmaceutical Sciences, Faculty of Life Sciences & Medicine, King’s College London, Franklin-Wilkins Building, 150 Stamford Street, London SE1 9NH, UK; driton.vllasaliu@kcl.ac.uk; Tel.: +44-020-7848-1728

Non-invasive drug delivery generally refers to painless drug administration methods involving drug delivery across the biological barriers of the mucosal surfaces or the skin. Non-invasive drug administration options offer obvious advantages over injection; they are preferred by the patients and could result in improved access to medication and adherence to therapy, as well as achieve improvement in the drug safety profile. Furthermore, non-invasive drug administration avoids needle-related complications and the high healthcare costs associated with drug administration by healthcare professionals. However, many drugs, including many small drug molecules and almost all biologics, do not possess the ideal physicochemical properties that enable satisfactory and clinically relevant absorption from mucosal surfaces or the skin. Considering biologics in particular, this diverse class of highly effective drugs (e.g., peptides, therapeutic proteins, antibodies and nucleic acid-based therapeutics) has rapidly proliferated but drug administration options currently largely remain limited to injection. Therefore, the need for non-invasive drug delivery options is clear.

Non-invasive drug delivery research is an active, rich and multidisciplinary research area and this Special Issue aims to present the current state of the art in the field. The contributions in this Special Issue address drug delivery systems for administration through the oral route [1,2,3,4], respiratory administration [5,6], nose-to-brain delivery [7] and via the skin [8,9,10,11,12,13,14]. Furthermore, a broad range of drug delivery systems and therapeutics (both small drug molecules and biologics) for non-invasive delivery were investigated and discussed, including nanosystems such as silica nanoparticles [15].

Utilising in vitro, ex vivo and in vivo studies, McCartney at al. [1] evaluated sucrose laurate (SL)—a food grade surfactant—as an intestinal permeation enhancer (PE). This comprehensive mechanistic study demonstrated that SL increased macromolecular permeability in vitro (intestinal Caco-2 cells), ex vivo (rat colonic tissue) and in vivo (rat jejunal and colonic instillations) via a mechanism that involved plasma membrane perturbation, leading to tight junction opening and facilitating paracellular flux. However, the study highlights the fine line between toxicity (sub-lethal toxic effects were determined by high content analysis) and potential application as a PE, which is a typical challenge encountered in these types of studies. Carobolante et al. [2] report on the potential utility of extracellular vehicles (EVs) for improving intestinal drug permeability. This study compared EVs isolated from bovine milk with those derived from intestinal epithelial cells. While epithelial cell-derived EVs showed a superior effect, milk-derived systems also improved the cell uptake and intestinal permeability (in vitro) of a model drug which otherwise showed poor permeability. The study therefore highlighted the potential of these safe ‘biological nanoparticles’, which can be derived from inexpensive and sustainable sources, in oral drug delivery. Bahman et al. [3] report the development of a poly-(styrene-co-maleic acid) (SMA) micellar system for oral insulin delivery. These systems were shown to improve the permeation of insulin across the Caco-2 intestinal cell model in vitro, showed efficient insulin permeation across an ex vivo intestinal (rat) model and produced hypoglycaemic effects in healthy and diabetic mice. New [2] provides an overview of the biological mechanisms which have been exploited to improve oral delivery of biologics, providing examples and an update on the latest clinical trials in the area.

Alabsi et al. [5] report on the successful design of spray-dried inhalable dry powders containing the glycopeptide MMP2200 (lactomorphin)—a novel glycopeptide opioid agonist—either alone or in combination with the excipient trehalose for intranasal and deep lung administration. A comprehensive physicochemical characterisation was reported, concluding that the formulations possessed the required properties for respiratory drug delivery as inhaled dry powder aerosols. Liang et al. [6] discuss some of the challenges and strategies in the formulation of biologics for pulmonary delivery, together with the recent clinical developments in inhaled biologics for local and systemic delivery. Froelich et al. [7] illustrate the potential utility of nasal administration for drug delivery to the brain by summarising the current status of research related to microemulsion-based systems for nose-to-brain delivery.

Articles focusing on delivery via the skin report a range of delivery systems. This includes sodium alginate and poly(vinyl) alcohol hydrogels for enhanced skin delivery of quercetin (a natural polyphenolic flavonoid commonly found in fruits and vegetables and known for anti-inflammatory and antioxidant effects) as an example system that could be considered as an option for the treatment of skin ageing and inflammation [8]. Microneedle-based systems for administration of local anaesthesia (lidocaine) are reported by Yang et al. [9], confirming the delivery of clinically-relevant levels of lidocaine and a fast onset time. Akrawi and colleagues [10] detail the generation of a chitosan-coated nanoemulsion system for delivery of naringenin (a natural flavonoid known to possess antioxidant, antimicrobial and anti-inflammatory characteristics) as a potential platform to accelerate wound healing. A non-invasive system for transdermal vaccine delivery is highlighted by Kitaoka et al. [11], whereby the system was based on the skin penetration enhancer, monoolein, and an antigen model protein (ovalbumin) was delivered to immune cells in living mice, inducing antigen-specific IgG antibodies. Work by Nunes et al. [12] reports topical formulations for the management of psoriasis based on new and modified neutrophil elastase inhibitors loaded in topical emulsion and microemulsion formulations. Finally, Molinaro et al. [13] describe multidrug ultradeformable vesicles for the treatment of skin inflammation, reporting that Tween^®^ 80-containing systems showed interesting properties in terms of skin permeation and drug release profiles, as well as in vivo anti-inflammatory activity.

Overall, the articles of this Special Issue highlight that this field is very active, and progress in the understanding of the biological barriers and materials is being harnessed in the development of novel strategies for non-invasive drug delivery.

## Data Availability

Not applicable.
